# Intratracheal Aerosolization of *Nocardia farcinica* in Mice Optimizes Bacterial Distribution and Enhances Pathogenicity Compared to Intranasal Inoculation and Intratracheal Instillation

**DOI:** 10.3390/biom15070950

**Published:** 2025-06-30

**Authors:** Bingqian Du, Ziyu Song, Jirao Shen, Jiang Yao, Shuai Xu, Xiaotong Qiu, Min Yuan, Zhenjun Li

**Affiliations:** 1 National Key Laboratory of Intelligent Tracking and Forecasting for Infectious Diseases, National Institute for Communicable Disease Control and Prevention, Chinese Center for Disease Control and Prevention, Beijing 102200, China; 2112041038@stu.gdpu.edu.cn (B.D.); songziyu0923@gmail.com (Z.S.); shenjirao0828@gmail.com (J.S.); yaojiang0919@gmail.com (J.Y.); xushuai@icdc.cn (S.X.); qiuxiaotong@icdc.cn (X.Q.); yuanmin@icdc.cn (M.Y.); 2 Wenzhou Key Laboratory of Sanitary Microbiology, Key Laboratory of Laboratory Medicine, Ministry of Education, School of Laboratory Medicine and Life Sciences, Wenzhou Medical University, Wenzhou 325035, China

**Keywords:** *Nocardia farcinica*, intratracheal aerosolization, mice pneumonia model

## Abstract

*Nocardia*, an easily missed but potentially fatal opportunistic pathogen, can lead to serious infections like lung and brain abscesses. Intranasal inoculation (IN) is the traditional approach for constructing a *Nocardia*-induced pneumonia mice model, while it usually only results in limited local bacterial infection in the lungs. To comprehensively assess infection dynamics across distinct pulmonary inoculation routes in mice models, this study compared the pathogenicity of three different *Nocardia farcinica* pneumonia models established via IN, intratracheal aerosolization (ITA), and intratracheal instillation (ITI). C57BL/6J mice were infected with *N. farcinica* through IN, ITA and ITI with comparative analyses of bacterial distribution in lungs, survival rate, weight, bacterial load, inflammatory cytokines, histopathological characteristics and transcriptome differences. The findings suggest that ITA *N. farcinica* infections caused severer clinical symptoms, higher mortality, pulmonary bacterial load, levels of inflammatory cytokines in bronchoalveolar lavage fluid, and more significant histopathological damage to lungs than IN and ITI. Furthermore, ITA resulted in better lung bacterial distribution and delivery efficiency than ITI and IN. Transcriptome analysis of lungs from *N. farcinica* infected mice via IN, ITA and ITI revealed significant differential gene expression, whereas ITA route resulted in a larger fold change. ITA provides a more consistent and severe model of *N. farcinica* pneumonia in mice than IN and ITI, which can make the bacteria more evenly distributed in the lungs, leading to more severe pathological damage and higher mortality rates. In conclusion, ITA is an optimal route for developing animal models of *N. farcinica* pneumonia infections.

## 1. Introduction

*Nocardia* is a Gram-positive, facultative aerobic, intracellular parasitic, actinomycetes, and nocardiosis is one of the highly lethal opportunistic disease. It is mainly infected immunocompromised patients, often causing severe infections such as pneumonia, brain abscess, meningitis, bacteraemia, skin and soft tissue infection and keratitis [1,2]. However, immunocompetent individuals are also at risk of *Nocardia* infection [3]. In recent years, with the growing number of elderly individuals, immunosuppressed patients, organ transplant patients and human Immunodeficiency Virus (HIV) patients, the infection rate of *Nocardia* has gradually increased [4,5]. About 80% of patients with *Nocardia* infection are caused by inhaling aerosols containing *Nocardia* through the respiratory tract [6,7]. If pulmonary nocardiosis is not treated promptly and effectively, it is highly likely to spread to other parts of the body, causing more serious consequences [8]. Our previous research found that the all-cause mortality rate of patients with *Nocardia* infection is approximately 20%. Still, other research has shown that the mortality rate of patients with *Nocardia* bacteremia is approximately 50% [9], and the patients with disseminated infections range from 44% [10] to 85% [11]. *Nocardia* infection, as a fatal infection, faces severe clinical challenges.

Individuals infected *Nocardia farcinica* is widely distributed in Asia, Europe and North America and one of the most common clinical pathogenic bacteria among *Nocardia* species [12]. *N. farcinica* can invasively destroy the lungs and even the brain, leading to severe lung infections, brain abscesses, and so forth [13]. Our previous studies expressed that *N. farcinica* accounts for over a quarter (28%) of the brain *Nocardia* infectious cases and 14% of all *Nocardia* infectious cases, moreover, since 2000, the number and proportion of *N. farcinica* cases have been trending upwards [5]. Accordingly, *N. farcinica* is considered the most pathogenic species within the *Nocardia* species due to its wide distribution, invasiveness and high morbidity [14].

Reliable mice models are solid foundations for characterizing the invading *Nocardia* and the host response, and assessing the efficacy of new antibiotics. Our laboratory has already constructed a *Nocardia* bloodstream infection model in the early stage, which can effectively simulate the infection process of *Nocardia* invading the brain from the blood, leading to brain infection [15]. This contributes to the study of the mechanisms related to *Nocardia*’s invasion of the blood-brain barrier. While established pneumonia models utilizing Swiss Webster mice via intranasal inoculation (IN) exist [16], *Nocardia* distribution in mice lungs is heavily influenced by the instilled volume and the degree of anaesthesia [17]. Furthermore, this infection route are inconsistent with the progression of the disease in humans. A recent study suggests that intratracheal instillation (ITI) with *Acinetobacter baumannii* results in a more sustained lung infection compared to IN [17]. Another study demonstrates that intratracheal aerosolization (ITA) is a better method for modelling influenza virus-induced pneumonia in mice than IN, as it can lead to more severe lung injury and higher viral loads in the lungs [18]. In addition, Verma et al. highlighted that non-invasive aerosol infections are convenient and cost-effective, noting that aerosol exposure to *Mycobacteroides abscessus* closely mimics natural airborne infections [19]. The fact is ITI and ITA permit the precise administration of bacterium or virus directly into the lung [17,18,19]. Therefore, we hypothesized that ITA or ITI would yield more uniform pulmonary distribution and higher pathogenicity than IN in mice pneumonia model by *N. farcinica*.

Here, C57BL/6J mice were employed to construct a *N. farcinica* pneumonia model in this study. The purpose was to compare the different pathogenic phenotypes, disease progression, pulmonary bacterial load, inflammatory cytokines, and histopathological characteristics from three infection routes (IN, ITI and ITA) in the mice model. Additionally, mechanisms that may contribute to these observed variances were investigated by transcriptome sequencing method. This method can reveal dynamic changes in host specific gene expression during pathogen infection, enhancing our understanding of the pathogenesis.

## 2. Materials and Methods

### 2.1. Bacterial Strain

*N. farcinica* IFM 10152 was from the German Resource Centre for Biological Materials, Brunswick, Germany. *N. farcinica* IFM 10152 was isolated from the bronchus of an elderly male patient in Japan [20].

### 2.2. Animals and Ethic Statement

Pathogen-free female C57BL/6J mice (SPF Biotechnology Co., Ltd., Beijing, China), 6–8 weeks old, were given 7 days for acclimation after arrival. The mice were kept under a 12-h light-dark cycle and were provided rodent feed (SPF Biotechnology Co., Ltd., Beijing, China) and filtered tap water ad libitum in a sterile environment. Mice were monitored and recorded for clinical signs (Table S1), survival rate and daily weight changes throughout the entire period of the studies. The weight was measured using an electronic balance (YH-C2003, Yingheng Intelligent Equipment Co., Ltd., Dongyang, China) with weight readings accurate to one thousandth of a gram. All animal experiments were approved by the ethics review committee of the Chinese Center for Disease Control and Prevention, and the ethical approval number was 2024-049.

### 2.3. Inoculum Preparation for Lung Infection

The day before infection, *N. farcinica* IFM 10152 was inoculated into Brain Heart Infusion (BHI) broth (Oxoid Ltd., Hants, UK) and cultivated overnight at 37 °C with continuous shaking at 180 rpm. The subsequent overnight culture was diluted with BHI at 1:100 and incubated at 37 °C with shaking until reaching logarithmic growth phase. Subsequently, the final cultures were subjected to centrifugation, followed by washing, resuspension and adjustment in phosphate-buffered saline (PBS) [Thermo Fisher Scientific, Waltham, MA, USA] to achieve concentrations of bacterial suspensions 1 × 10^6^ colony-forming units (CFU), 1 × 10^7^ CFU, 1 × 10^8^ CFU in 50 µL, respectively.

### 2.4. Mice Models of Pneumonia

**Figure 1:**
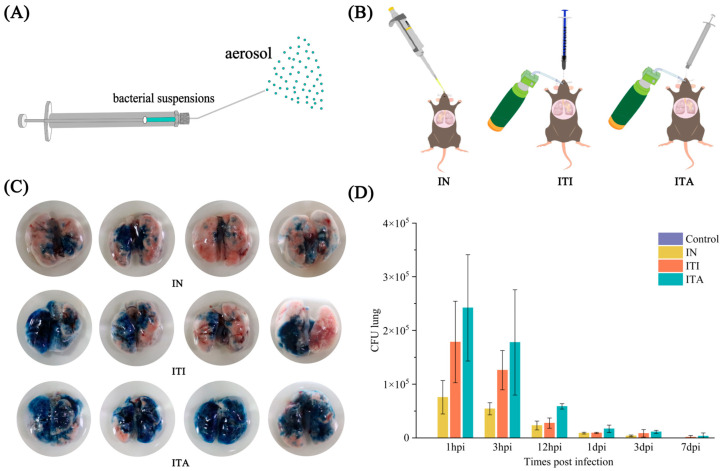
Characterization of *Nocardia farcinica* aerosolization. (**A**) Physical diagram of MicroSprayer. (**B**) Schematic diagrams of intranasal inoculation (IN), intratracheal instillation (ITI) and intratracheal aerosolization (ITA). (**C**) Trypan Blue staining solution was inoculated into mice lungs by IN, ITI and ITA, respectively (4 mice per group). (**D**) The bacterial load in bronchoalveolar lavage fluid (BALF) of mice infected with *N. farcinica* IFM 10152 in different routes at each point of time (5 mice per group per time point).

Mice were randomly segregated into nine infection groups and one PBS control group (seven mice per group). The infection groups were challenged with 1 × 10^6^ CFU, 1 × 10^7^ CFU, and 1 × 10^8^ CFU *N. farcinica* suspensions in 50 µL via IN, ITI, and ITA routes, respectively. Our previous research confirmed that control groups for different infection routes had no significant effect on mice [15], so the control group randomly received three infection routes of the same volume of PBS as the infection group. Regardless of the infection route, mice were anesthetized with pentobarbital sodium first. IN: *N. farcinica* suspensions (50 µL) were slowly released into mice nostrils with a 100 microliter pipette (Eppendorf AG, Hamburg, Germany) to avoid the formation of bubbles. ITI: Mice Upper incisors were hanged on nylon ropes on a tilted small animal operating table (HRH-HAG6, Huironghe Company, Beijing, China), and a small animal laryngoscope (HRH-MAG5, Huironghe Company, Beijing, China) was inserted into their deep oral cavity. Once the tracheal opening was observed using the small animal laryngoscope, the delivery tube with 50 µL *N. farcinica* suspensions was inserted into their trachea for injection. ITA: *N. farcinica* aerosolization was accomplished with a MicroSprayer (HRH-MAG4, Huironghe Company, Beijing, China) (Figure 1A and Figure S1). Using the same method, mice were fixed onto a small animal operating table. Once the tracheal opening was observed using the laryngoscope, the MicroSprayer was inserted into their trachea for rapid injection to achieve aerosolization of *N. farcinica*. The delivery tube (ITI) and the MicroSprayer (ITA) both were inserted 25 mm from the larynx (near the tracheal bifurcation). The three infection routes were shown in Figure 1B.

For bacterial load (five mice per time point, 30 mice per group), inflammatory cytokines (five mice per time point, 30 mice per group), histopathological characteristics (three mice per time point, nice mice per group) and transcriptomics research (three mice per time point, six mice per group), mice were infected with 1 × 10^7^ CFU *N. farcinica* suspensions in 50 µL via three routes (mice allocation to each group was performed using randomization principles). Lung tissues and bronchoalveolar lavage fluid (BALF) were collected at the designated timepoints.

### 2.5. Aerosol Distribution of Trypan Blue

Anesthetized mice were fixed on the tilted small animal operating table. 50 µL of 0.4% Trypan Blue staining solution (Biosharp, Hefei, China) were inoculated into mice using the three infection routes (four mice per group) mentioned above. Following the injection of Trypan Blue staining solution, the mice were immediately euthanized and their lungs were extracted. The aim of using Trypan Blue staining solution was to compare the bacterial distribution effects of three infection routes in the lungs through visual observation.

### 2.6. BALF Collection and Bacterial Load Determination

BALF was collected at 1 h post infection (hpi), 3 hpi, 12 hpi, 1 day post infection (dpi), 3 dpi and 7 dpi, respectively. The BALF was obtained by instilling 1000 µL PBS pre cooled at 4 °C into the trachea of mice, followed by two repeated lavages. The collected BALF was transferred into 1.5 mL EP tubes. To quantify the *N. farcinica* bacterial load in BALF, serial dilutions of the homogenized BALF samples were plated on BHI agar and incubated at 37 °C for 48 h. Remaining BALF was centrifuged at 4 °C for 10 min at 1500 rpm. This BALF supernatant was then stored at −80 °C for measurement of inflammatory cytokines level.

### 2.7. Detection of Inflammatory Cytokines in Mice BALF

BALF cytokines-interleukin-4 (IL-4), interleukin-10 (IL-10), tumor necrosis factor-alpha (TNF-α), and interferon-gamma (IFN-γ)—were quantified by the Mouse IL-4 and IFN-γ Uncoated ELISA Kit (Thermo Fisher Scientific, Waltham, MA, USA) and the Mouse IL-10 and TNF-α ELISA Set Ⅱ (BD OptEIATM, San Diego, CA, USA), according to the manufacturer’s protocol and measured with the multi functional microplate detector (Bio Tek Instruments, Valemount, VT, USA).

### 2.8. Histopathological

Lungs were removed, fixed in 4% paraformaldehyde (Wuhan Servicebio Technology Co., Ltd., Wuhan, China) for at least 1 day, embedded in paraffin. Thereafter 5 μm-thick samples were sectioned and stained with Haematoxylin and Eosin (H&E). Subsequently, all samples were examined using a pannoramic confocal scanner (PANNORAMIC-DESK-MIDI-250-1000, 3DHISTECH Ltd., Budapest, Hungary) according to the manufacturer’s instructions. Pathological analysis was performed by the two pathologists (blind to infection). The histopathology score was mainly determined by evaluating the degree of inflammatory cell infiltration, alveolar wall thickening, haemorrhage, bruising, and perivascular edema through a 4-point scoring system (0, within normal limits; 1, minimal; 2, slight; 3, moderate; 4, severe) [21]. The inter-rater agreement between the two pathologists showed excellent reliability, with a Cohen’s kappa value of 0.81 and a coefficient of variation (CV) below 10%.

### 2.9. RNA Extraction, Library Preparation, Sequencing and Analysis

TRIzol Reagent (Invitrogen, Carlsbad, CA, USA) was used to extract total lung RNA at 1 and 3 dpi from *N. farcinica*-infected mice and PBS controls (three replicates per group). Then RNA quality was assessed by a Bioanalyser (Agilent 5300, Agilent, Palo Alto, CA, USA) and purity was measured by the NanoPhotometer (ND-2000, NanoDrop Technologies, Seattle, WA, USA). After confirming RNA quality, mRNA enrichment and purification were performed using magnetic beads with oligo (dT). Double-stranded cDNA was synthesized using a SuperScript double-stranded cDNA synthesis kit (Invitrogen, Carlsbad, CA, USA) with random hexamer primers. After quantified by Qubit 4.0, the sequencing library was performed on the NovaSeq X Plus platform (PE150) using NovaSeq Reagent Kit at Shanghai Majorbio Bio-pharm Biotechnology Co., Ltd. (Shanghai, China). The raw paired-end reads were trimmed and quality-controlled using fastp with default parameters [22]. Subsequently, the clean reads were separately aligned to reference genome with orientation mode using HISAT2 2.2.0 software [23]. After quantifying gene abundances by RSEM 1.3.3 [24], differential expression analysis was performed using the DESeq2 1.36.0 [25].

### 2.10. Statistical Analysis

Origin software (OriginLab, Northampton, MA, USA) was used for data analysis and visualization. Data were presented as means and standard deviations. Survival curves were compared using the log-rank (Mantel-Cox) test. For each challenge dose, a global log-rank test was first performed across all four groups, followed by pairwise comparisons between groups with *p*-values adjusted for multiple comparisons using the Benjamini-Hochberg method. The weight changes were used a linear mixed effects model (LMM) analysis. Student’s *t*-test was used to analyze between each experimental group and the PBS control group. Analysis of variance (ANOVA) was used to determine BALF cytokines. Differential transcriptome and functional enrichment were analyzed on the online platform of Majorbio Cloud Platform [www.majorbio.com (accessed on 2 March 2025)], and Gene Ontology (GO) and KEGG charts were generated [26]. The protein-protein interaction network was visualized using Cytoscape 3.10.3 software (Institute of Systems Biology, Seattle, WA, USA) (* *p* < 0.05; ** *p* < 0.01; *** *p* < 0.001).

## 3. Results

### 3.1. Mice Infected with N. farcinica via ITA Route Exhibit More Uniform Distribution in Lungs

Wang J et al.’s research shows that the aerosol particles sprayed by the MicroSprayer are fine enough to allow bacteria to be deposited in the deep lung regions [27]. To assess the bacterial distribution effects within the lungs under different infection routes, C57BL/6J mice were inoculated with Trypan Blue staining solution. As shown in Figure 1C, Trypan Blue staining solution is mainly located at the junction between the lungs and bronchi via the IN route. Although the ITI route can sometimes lead to an even distribution of staining solution in the lungs, it can also result in staining solution being distributed on only one side of the lung, lacking stability. This variability in ITI distribution may result from inconsistent flow direction of the bacterial suspension near the tracheal bifurcation. The results indicated that ITA route was more effective and stable in penetrating into the lungs of the mice and achieving uniform particle distribution compared to IN and ITI.

### 3.2. Mice Infected with N. farcinica via ITA Route Results in Higher Fatality, More Weight Loss and More Severe Bacterial Burden

**Figure 2:**
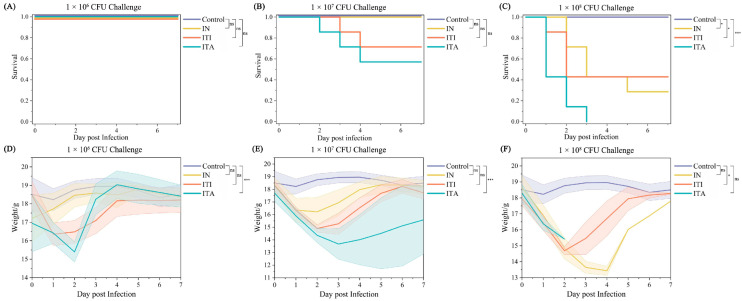
Survival and weight change curve plots for multi-dose *Nocardia farcinica* and phosphate-buffered saline (PBS). C57BL/6J mice (7 mice per group) were infected by intranasal inoculation (IN), intratracheal instillation (ITI) and intratracheal aerosolization (ITA) with 1 × 10^6^ Colony-forming units (CFU), 1 × 10^7^ CFU, and 1 × 10^8^ CFU *N. farcinica* suspensions in 50 µL PBS, respectively, and monitored daily for 7 days for survival (**A**–**C**) and weight (**D**–**F**) (ns: not significant, * *p*  <  0.05, *** *p*  <  0.001).

The effects of various doses on time to death and percent survival were showed in Figure 2A–C. At infectious doses of 1 × 10^6^ CFU in 50 µL, the survival rate all reached 100%. While at infectious doses of 1 × 10^8^ CFU in 50 µL, IN, ITI and ITA resulted in death rates of 71%, 57% and 100% (*χ*^2^ = 19.2, *p* < 0.001), respectively. At infectious doses of 1 × 10^7^ CFU in 50 µL, the survival rate of IN route reached 100%, but ITI and ITA resulted in death rates of 29% and 43% (*χ*^2^ = 3.6, *p* = 0.02). Weight loss was observed earlier and faster in the mice via ITA compared to those received IN or ITI when administered the same infectious doses (*F* = 11.527, 4.662, 3.424, respectively, all *p* < 0.05) (Figure 2D–F). In contrast, the PBS control remained stable throughout. Due to the fact that infectious doses of 1 × 10^6^ CFU in 50 µL were non lethal in all routes and 1 × 10^8^ CFU in 50 µL was lethal in the ITA, our subsequent studies will select dose of 1 × 10^7^ CFU in 50 µL. As seen in Figure 1D, ITA infection resulted in a higher bacterial load in lungs compared to the other two infection routes. And bacterial load in lungs of mice infected via different routes peaked at 1 hpi.

### 3.3. Mice Infected with N. farcinica via ITA Route Exhibit More Severe Clinical Symptoms and Inflammatory Responses

**Figure 3:**
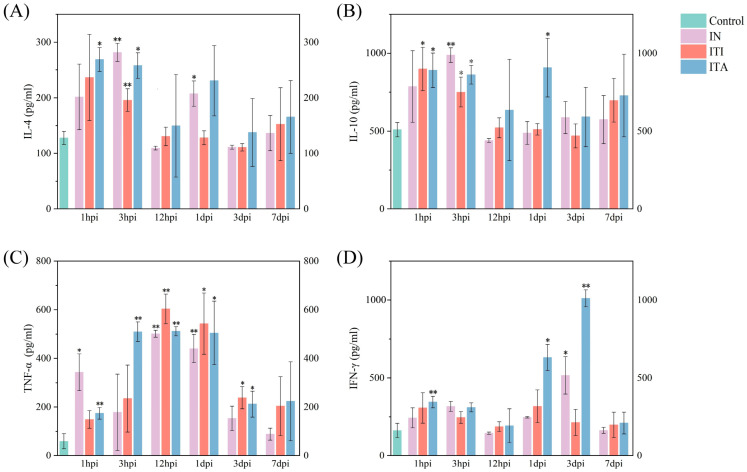
Bronchoalveolar lavage fluid (BALF) inflammatory cytokines of C57BL/6J mice infected with *Nocardia farcinica* IFM 10152 in different routes at each point of time (5 mice per group per time point). Mice were infected for one time via intranasal inoculation (IN), intratracheal instillation (ITI) and intratracheal aerosolization (ITA) with 1 × 10^7^ Colony-forming units (CFU) in 50 µL Phosphate-buffered saline (PBS), respectively. The data of PBS group was acquired from the mice infected with 50 µL PBS at 1 hpi. Inflammatory cytokine levels, including (**A**) Interleukin-4 (IL-4), (**B**) Interleukin-10 (IL-10), (**C**) Tumor necrosis factor-alpha (TNF-α), and (**D**) Interferon-gamma (IFN-γ), in BALF were measured by ELISA. Student’s t test was conducted between each experimental group and the PBS control group (* *p*  <  0.05, ** *p*  <  0.01).

As far as clinical monitoring is concerned, all mice infected with *N. farcinica* showed piloerection of hair on back, reduced food and water intake and emaciation at 1 dpi (Figure S2A). At 1 dpi, the mice infected with *N. farcinica* via ITA route showed skin lesions of back (Figure S2B). At 2 dpi, the mice infected with *N. farcinica* via ITI route showed eyes not fully open (Figure S2C) and the mice infected with *N. farcinica* via ITA route showed eyes half closed or more with secretions (Figure S2D). Furthermore, the mice infected with *N. farcinica* via ITA showed anal blockage with feces and difficulty in defecation (Figure S2E). As far as clinical scores, the mice infected with *N. farcinica* via ITA showed higher score since severe emaciation, moderate piloerection, marked reduced motor activity, slightly decreased respiration and eyes half closed or more (Table S2). As shown in Figure 3, IL-4 and IL-10 levels dramatically increased in the ITI and ITA infection group at 1 hpi, but significantly increased in the IN infection group at 3 hpi. TNF-α expression levels was significantly upregulated in all three infection groups at 12 hpi and 1 dpi. IFN-γ levels was significantly increased in the IN and ITA infection group at 3 dpi, no significant difference in expression was observed in the ITI group.

### 3.4. ITA Route Leads to More Pronounced Histopathological Lesion

**Figure 4:**
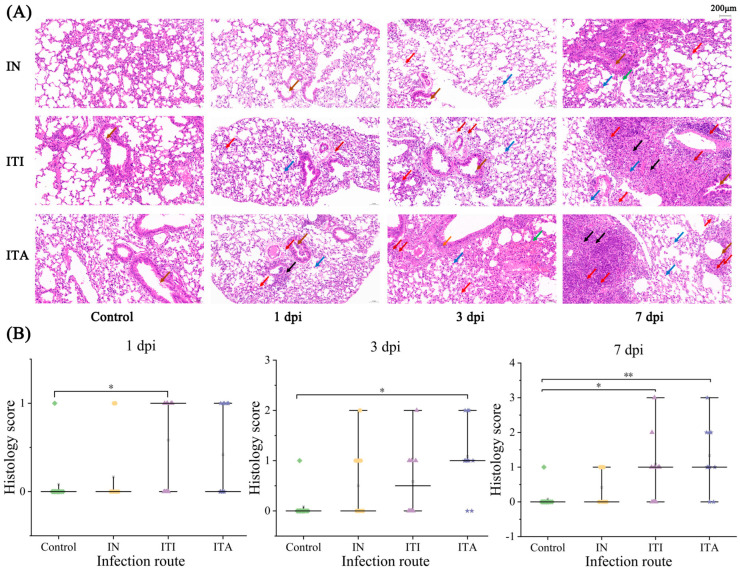
Pathological analyze and pathological score in mice at 1, 3 and 7 dpi (3 mice per group per time point). (**A**) Histopathological characteristics and (**B**) histopathology score of lung tissue in infected mice at 1, 3 and 7 dpi under 200× magnification. Arrows indicated lesions: brown for eosinophilic material visible in the lumen of the alveoli; red for inflammatory cell infiltration; dark red for lymphocyte andgranulocyte infiltration; blue for alveolar macrophage infiltration; black for necrotic cellular debris of lung tissue; green for perivascular bleeding; orange for cytoplasmic looseness of bronchial epithelial cells (* *p*  <  0.05, ** *p*  <  0.01).

To compare the extent of histopathological damage caused by three different infection routes, the left lungs of mice were removed at 1, 3 and 7 dpi. Histopathological analysis showed no significant inflammation or abnormalities in the PBS control group. No pathological changes were observed in the lungs at 1 dpi following infection via IN; however, slight granulocyte infiltration in the alveolar wall and macrophage infiltration in the alveolar cavity were noted at 3 and 7 dpi, small scale alveolar hemorrhage were noted at 7 dpi. In the ITI and ITA group, large areas of granulocyte infiltration, macrophage infiltration and lymphocytic infiltration in the alveolar wall or alveolar cavity were observed, accompanied by multiple connective tissue hyperplasia and necrotic cellular debris. In addition, small scale alveolar hemorrhage in the alveolar cavity and perivascular, and a few cytoplasmic looseness of bronchial epithelial cells were noted in the ITA group. Histopathological scores were higher after ITA infection than IN and ITI infection at the three time points (Figure 4).

### 3.5. Differential Gene Expression Analysis

**Figure 5:**
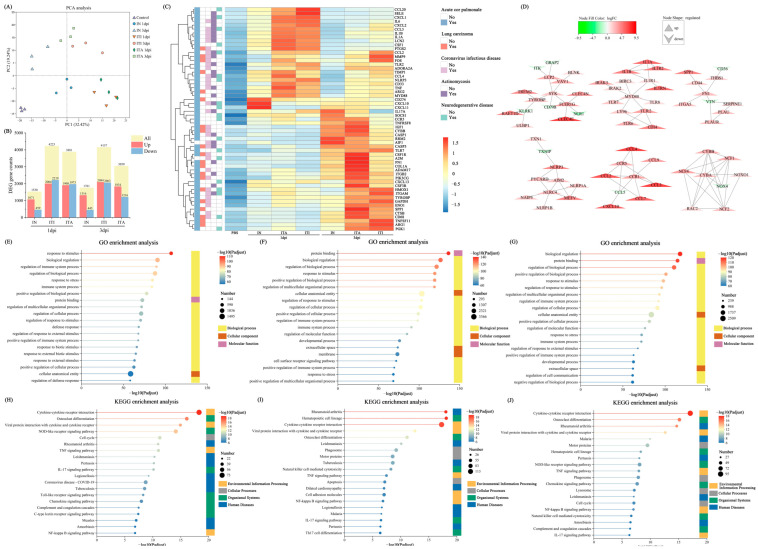
Differentially expressed genes (DEGs) in *Nocardia farcinica*-infected C57BL/6J mice after intratracheal aerosolization (ITA) obtained by RNA-seq compared to intranasal inoculation (IN) and intratracheal instillation (ITI). (**A**) Principal component analysis (PCA) of normalized RNA sequencing results for each infection route. (**B**) Numbers of DEGs in each infection route. (**C**) Heatmap displayed the expression patterns of 57 differential genes associated to 5 related diseases. (**D**) The protein-protein interactions of 3 dpi DEGs [ITA vs Phosphate-buffered saline (PBS) control] were analyzed using Cytoscape software. GO analysis of lung tissues infected with *N. farcinica* via (**E**) IN, (**F**) ITI, and (**G**) ITA at 3 dpi. DEGs KEGG pathway analysis of lung tissues infected with *N. farcinica* via (**H**) IN, (**I**) ITI, and (**J**) ITA at 3 dpi.

To further explore the different mechanism in transcript levels among IN, ITI and ITA administration of *N. farcinica*, the lung transcriptome of C57BL/6J mice infected with *N. farcinica* was determined at 1 and 3 dpi. Mice that received 50 µL PBS were designated as the control group. Principal component analysis (PCA) was conducted using normalized counts to evaluate the quality of the transcriptome data (Figure 5A). Lung transcriptome data from different infection routes at 1 and 3 dpi were compared with the PBS control group to identify differentially expressed genes (DEGs) (differential expressed genes with |log_2_fold change| ≥ 1 and adjusted *p* <  0.05 were considered statistically significant). The total number of DEGs in each comparison group is shown in Figure 5B. The diagram indicated that compared to IN, the DEGs increased after ITI and ITA infection.

Transcriptomic analysis r evealed that mice infected *N. farcinica* via three infection routes led to differential gene expression patterns associated with diseases related to lung and brain infections, including acute cor pulmonale, lung carcinoma, Coronavirus infectious disease, Actinomycosis and neurodegenerative disease. We analyzed DEGs related to the above-mentioned diseases and generated a heatmap (Figure 5C). In comparison to the IN infection group, the ITA and ITI infection groups resulted in a significant up-regulation of genes in the lungs. Many up-regulated genes are closely related to inflammation and immune responses, such as CCL20, IL6, CXCL1, CSF1R, CCR5, TNFRSF8, indicating that mice infected *N. farcinica* via these two infection routes may lead to robust inflammatory response. Nevertheless, there seem to be different differential gene expression patterns at different time points. Specifically, the DEGs at 1dpi are closely related to immune inflammation and tissue repair, while the genes at 3dpi are mostly associated with disease status. Still further, ITA route seems to have resulted in more significant gene up-regulation. In addition, we conducted protein-protein interaction (PPI) analysis on DEGs between the ITA infection group and the PBS control group during *N. farcinica* infection (Figure 5D). Among the DEGs in this two groups, MYD88, CCR1, SYK, CYBB, and CYBA might play important roles in the progression of *N. farcinica* infection.

GO analysis revealed that of the top 20 enriched terms following *N. farcinica* infection via three routes, the primary activations were related to immune processes and cellular process (Figure 5E–G). Significant enrichment was observed in response to stimulus, biological regulation, protein binding, cellular anatomical entity after *N. farcinica* infection through three routes. However, there are more DEGs enriched in the ITI and ITA infection groups compared to IN group, and corresponding *P*-adjusted are more significant.

Additionally, KEGG pathway enrichment analysis revealed significant differences in immune response, disease-related pathways, and inflammation response under different infection routes (Figure 5H–J). The ITA and ITI infection groups exhibited more significant natural killer cell-mediated cytotoxicity and Th17 cell-mediated immune response, indicating that these two infection routes play important roles in immune killing and inflammatory responses. Notably, KEGG pathways of cell apoptosis, intercellular interactions, phagocytosis were highly enriched in the ITA and ITI infection groups, while the IN group were cell proliferation.

## 4. Discussion

Nocardiosis is one of the highly lethal opportunistic disease, causing pulmonary, brain and even systemic infections. These infections often lead to prolonged hospitalization and high medical expenses. Therefore, there is an urgent need to develop stable and reliable animal models to explore pathogenesis and novel therapeutics. Most cases of *Nocardia* infection are caused by inhaling aerosols that contain *Nocardia* [6,7]. There is no evidence supporting the notion that either of the two infection routes (IN and ITI) more accurately reproduces the human disease. In this study, we aimed to compare the use of ITA to IN and ITI in the ability to induce a robust pneumonia model of infection with *N. farcinica* IFM10152 in C57BL/6J mice. The results showed that *N. farcinica* infections via ITA caused more sever clinical symptoms, higher levels of inflammatory cytokines, and more significant histopathological damage than IN and ITI. Additionally, ITA resulted in better lung bacterial distribution, bacterial load, mortality and delivery efficiency than ITI and IN. ITA appears to be a more robust infection route for modelling *N. farcinica* pneumonia in mice.

ITA infection caused sever infection with obvious clinical symptoms, such as severely reduced respiration, impaired activity, emaciation, eyes half closed or more with secretions, and difficulty in defecation. And the ITA infection group showed more significant upregulation of cytokines (IL-4, IL-10, TNF-α, IFN-γ), histopathological damage to lungs, and higher bacterial load and mortality than the IN and ITI groups. This may be due to differences in the initial site of infection [18]. With IN route, *N. farcinica* initially occurred in the nasal cavity and subsequently spread to the lungs via respiratory tract. That implies that a reduced load of bacteria reaches the distal lungs via IN. While ITA and ITI routes ensured greater direct exposure of *N. farcinica* to the lungs bypassing the spreading process. Additionally, ITI infection route may lead to unilateral distribution of bacteria in the lungs, in contrast, ITA infection route performed more uniform lung distribution, pathologic homogeneity, and stable reproducibility among mice. Moreover, compared to being easily choked to death during the IN infection process, ITA seems to be safer and more effective. Furthermore, IFN-γ is a critical cytokine that regulates macrophage activation, playing a crucial role in host defense against pathogen invasion by triggering multiple antimicrobial effector functions and likely contributing significantly to the immune response against *N. farcinica* infection [28]. Further study may be needed to delineate the role of IFN-γ.

Transcriptome analysis revealed that the positive regulation of innate immunity and tissue repair were essential in the early stages of *N. farcinica* infection in mice. Compared to IN, ITA and ITI induced a stronger innate immune response. Interestingly, DEGs associated with neurodegenerative diseases, such as NLRP3, CSF1, and A2M, exhibit higher expression in the ITA infection group. These findings are consistent with the results of our previous *Nocardia* bloodstream infection model [15] and may indicate that ITA infection may simulate the process of human *Nocardia* infection very well. Specifically, when the host inhales aerosols containing *Nocardia*, lung infection first occurs and gradually spreads to the brain from the lungs [6,7,8,29]. In addition, we found that tumor related genes, such as PTGS2, ADAM17 and FOS, are highly expressed in the ITA infection group, suggesting that ITA may lead to more severe *N. farcinica* infections.

PPI analysis indicated that MYD88, CCR1, SYK, CYBB, and CYBA might play important roles in the progression of *N. farcinica* infection. Our previous research has also confirmed this finding, that the NbtS protein of *Nocardia* may activate the toll-like receptor 4 (TLR4) dependent MyD88, then activates MAPK and NF-κB signaling pathways and promotes inflammatory response [30]. However, the causal relationship between these DEGs and the progression of *Nocardia* infection has not been validated through corresponding experiments. In addition, it has been described in literature that elevated expression of SYK in *Nocardia* seriolae-infected largemouth bass [31], which may be related to phagocytosis of leukocytes [32]. Other genes have not been studied yet. Further studies will be required to understand these genes and their related pathogenic mechanisms.

The GO analysis revealed that the host mounts a robust immune response, regardless of infection route. KEGG pathway enrichment analysis further revealed significant differences in immune response and disease-related pathways, emphasizing the important element of immune response and cellular signal transduction to *N. farcinica* infection. Notely, the enrichment of pathways such as “Cytokine-cytokine receptor interaction”, “Natural killer cell mediated cytotoxicity” and “Phagosome” in the ITA and ITI groups further supports the idea that these routes may trigger a more aggressive immune response [18].

Additionally, this study still has some limitations. Firstly, we only used BALF to evaluate inflammatory cytokines, the lack of lung tissue homogenate CFU data at later time points limits our ability to comprehensively characterize established infection, and future studies could include tissue homogenate analyses. Secondly, we focused on assessing bacterial load and related outcomes in the lungs, without investigating the dissemination of *Nocardia* to other organs, such as the brain, liver, spleen, or kidneys, which could further clarify systemic spread following lung infection. Thirdly, while ITA effectively models severe *N. farcinica* pneumonia in mice by ensuring uniform pulmonary distribution, its particle size and deposition patterns may not fully mimic natural human inhalation, which involves variable particle sizes and less controlled deposition. This could potentially overestimate pathogenicity compared to real-world exposure scenarios.

## 5. Conclusions

In conclusion, ITA infection leads to more severe pathological damage of lungs, higher bacterial loads in the lungs and higher mortality compared to IN and ITI, which may be associated with the initial site of infection and host innate immunity. ITA provides a more consistent and severe model of *N. farcinica* pneumonia in mice than IN and ITI.

## Data Availability

The data that support the findings of this study are available from the corresponding author, Zhenjun Li, upon reasonable request.

## Supplementary Materials

The following supporting information can be downloaded at: https://www.mdpi.com/article/10.3390/biom15070950/s1, Figure S1: Diagram of particle distribution via a MicroSprayer (A). Schematic of mice infected *Nocardia farcinica* via a non-invasive intratracheal aerosolization (ITA) route (B); Figure S2: Clinical symptoms of infected mice. (A) One days after *Nocardia farcinica* infection via three infection routes, the mice showed piloerection of hair on back and emaciation. (B) One days after *N. farcinica* infection via intratracheal aerosolization (ITA) route, the mice showed skin lesions of back. Two days after *N. farcinica* infection via intratracheal instillation (ITI) (C) and ITA(D) route, the mice showed eyes not fully open or eyes half closed or more with secretions. (E) Two days after *N. farcinica* infection via ITA route, the mice showed anal blockage with feces and difficulty in defecation; Table S1: Clinical scoring system. (Animals are monitored regularly for signs of infection and scored according to value reported in the table below); Table S2: Clinical scores of mice infected with *Nocardia farcinica* via three infection routes at different time points. Table S3: Top 10 GO terms that are significantly enriched in intratracheal aerosolization at 3 dpi. Table S4: Top 10 KEGG terms that are significantly enriched in intratracheal aerosolization at 3 dpi.

## Abbreviations

The following abbreviations are used in this manuscript:

IN	Intranasal inoculation
ITA	Intratracheal aerosolization
ITI	Intratracheal instillation
HIV	Human Immunodeficiency Virus
BHI	Brain Heart Infusion
PBS	Phosphate-buffered saline
USA	the United States of America
CFU	Colony-forming units
BALF	Bronchoalveolar lavage fluid
hpi	Hour post infection
dpi	Day post infection
IL-4	Interleukin-4
IL-10	Interleukin-10
TNF-α	Tumor necrosis factor-alpha
IFN-γ	Interferon-gamma
CV	Coefficient of variation
LMM	Linear mixed effects model
H&E	Haematoxylin and Eosin
ANOVA	Analysis of variance
GO	Gene Ontology
PCA	Principal component analysis
DEGs	Differentially expressed genes
PPI	Protein-protein interaction
TLR4	The toll-like receptor 4
