# Supplementary material for: Intratracheal Aerosolization of Nocardia farcinica in Mice Optimizes Bacterial Distribution and Enhances Pathogenicity Compared to Intranasal Inoculation and Intratracheal Instillation
Source: Biomolecules. 2025 Jun 30;15(7):950. doi: 10.3390/biom15070950 (PMC12292814; doi:10.3390/biom15070950)
Supplement: Supplementary file 1 [file biomolecules-15-00950-s001.zip › biomolecules-3699762-supplementary.pdf]

Supplementary Materials S1

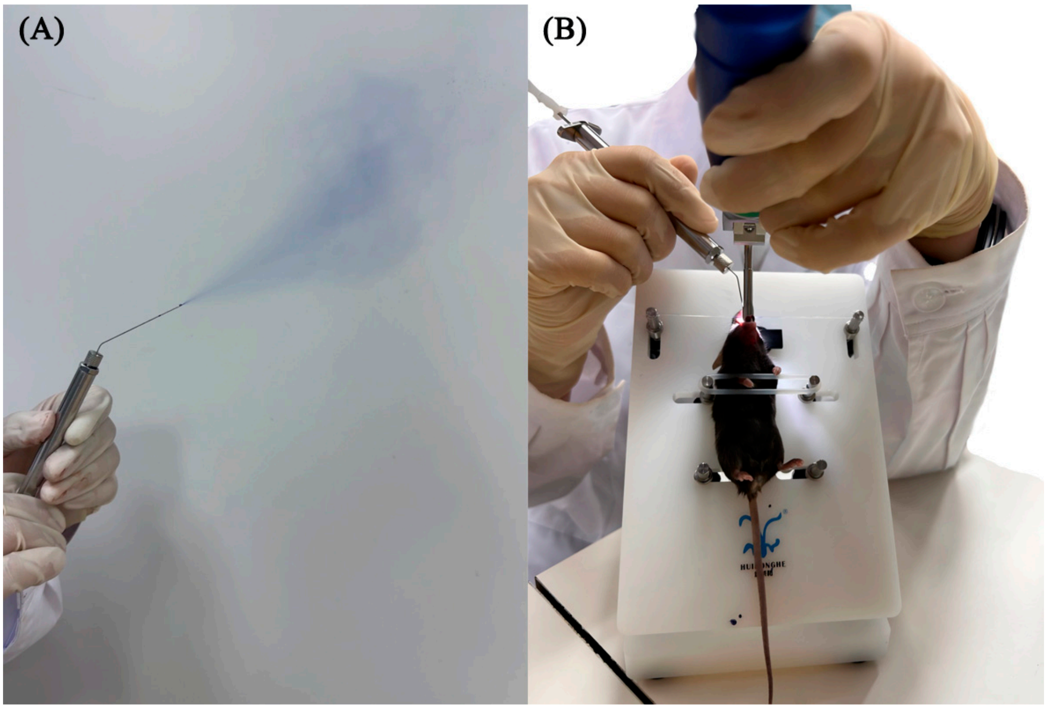

Figure S1. Diagram of particle distribution via a MicroSprayer (**A**). Schematic of mice infected *Nocardia farcinica* via a non-invasive intratracheal aerosolization (ITA) route (**B**).

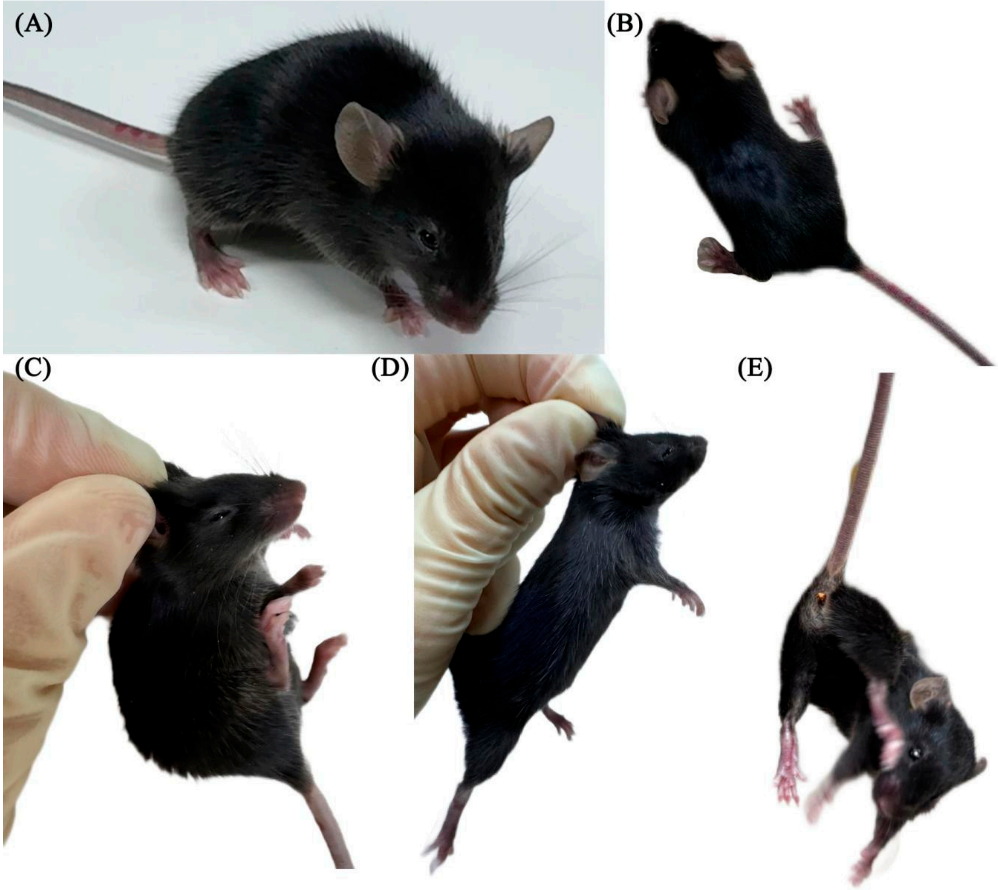

Figure S2. Clinical symptoms of infected mice. (**A**) One days after *Nocardia farcinica* infection via three infection routes, the mice showed piloerection of hair on back and emaciation. (**B**) One days

after *N. farcinica* infection via intratracheal aerosolization (ITA) route, the mice showed skin lesions of back. Two days after *N. farcinica* infection via intratracheal instillation (ITI) (**C**) and ITA(**D**) route, the mice showed eyes not fully open or eyes half closed or more with secretions. (**E**) Two days after *N. farcinica* infection via ITA route, the mice showed anal blockage with feces and difficulty in defecation.

Supplementary Materials S2

Table S1. Clinical scoring system. (Animals are monitored regularly for signs of infection and scored according to value reported in the table below).

Variable	Score and Description
Body weight [a]	0- weight lost <5%<br>1- weight lost 6–10%<br>2- weight lost 11–15%<br>3- weight lost 16–20%<br>4- weight lost >20%
Appearance	0- coat is smooth<br>1- patches of hair piloerected<br>2- majority of back is piloerected<br>3- piloerection, mouse appears "puffy"<br>4- piloerection, mouse appears emaciated<br>5- skin lesions, such as lump, ulcer
Activity	0- normal activity<br>1- slightly reduced activity<br>2- marked reduced activity<br>3- severely impaired activity
Posture	0- normal<br>1- slightly hunched, moving freely<br>2- hunched with activity<br>3- hunched without activity<br>4- ventral/lateral decubitus
Respiration rate	0- normal, rapid mouse respiration<br>1- slightly decreased respiration<br>2- moderately reduced respiration<br>3- severely reduced respiration<br>4- asphyxia
Eyes	0- open<br>1- eyes not fully open, possibly with secretions<br>2- eyes half closed or more, possibly with secretions<br>3- eyes closed or milky

[a] Body weight: percentage of body weight loss over time.

Table S2. Clinical scores of mice infected with Nocardia farcinica via three infection routes at different time points.

Time	Infection Routes	Variable								
		Mouse No.	1	2	3	4	5	6	7	Mean ± SD [e]
1dpi [a]	IN [b]	Body weight	0	0	1	1	0	0	0	0.29 ± 0.49
		Appearance	0	0	0	0	0	0	0	0.00
		Motor activity	0	0	0	0	0	0	0	0.00
		Posture	1	1	0	0	0	0	1	0.43 ± 0.53
		Respiration rate	0	0	0	0	0	0	0	0.00
		Eyes	0	0	0	0	0	0	0	0.00

		Total Scoring	1	1	1	1	0	0	1	0.71 ± 0.49
	ITI [c]	Body weight	0	1	0	1	1	1	0	0.57 ± 0.53
		Appearance	2	0	0	2	2	2	0	1.14 ± 1.07
		Motor activity	1	0	0	1	1	1	1	0.71 ± 0.49
		Posture	0	0	0	1	1	1	1	0.57 ± 0.53
		Respiration rate	1	1	1	1	1	1	1	1.00 ± 0.00
		Eyes	0	0	0	0	0	0	0	0.00
		Total Scoring	4	2	1	6	5	5	3	3.71 ± 1.80
	ITA [d]	Body weight	1	1	1	1	1	1	0	0.86 ± 0.38
		Appearance	5	4	2	4	2	2	2	3.00 ± 1.29
		Motor activity	2	2	1	1	2	2	1	1.57 ± 0.53
		Posture	3	3	3	3	3	1	3	2.71 ± 0.76
		Respiration rate	1	1	1	1	1	1	1	1.00 ± 0.00
		Eyes	0	0	0	0	0	0	0	0.00
		Total Scoring	9	11	8	10	9	7	7	8.71 ± 1.50
2dpi [a]	IN [b]	Body weight	0	1	0	0	0	0	0	0.14 ± 0.38
		Appearance	0	0	0	0	0	0	0	0.00
		Motor activity	0	0	0	0	0	0	0	0.00
		Posture	1	1	0	0	0	0	0	0.29 ± 0.49
		Respiration rate	0	0	0	0	0	0	0	0.00
		Eyes	0	0	0	0	0	0	0	0.00
		Total Scoring	1	2	0	0	0	0	0	0.43 ± 0.79
	ITI [c]	Body weight	0	1	0	0	1	0	0	0.29 ± 0.49
		Appearance	1	0	0	1	1	2	0	0.71 ± 0.76
		Motor activity	1	1	0	2	1	2	1	1.14 ± 0.69
		Posture	0	0	0	2	1	2	1	0.86 ± 0.90
		Respiration rate	1	1	1	1	1	2	1	1.14 ± 0.38
		Eyes	0	0	1	1	0	1	0	0.43 ± 0.53
		Total Scoring	3	3	2	7	5	9	3	4.57 ± 2.57
	ITA [d]	Body weight	1	-	1	1	1	0	1	0.83 ± 0.41
		Appearance	5	-	3	4	3	2	2	3.17 ± 1.17
		Motor activity	2	-	1	3	2	2	1	1.83 ± 0.75
		Posture	2	-	2	3	3	1	2	2.17 ± 0.75
		Respiration rate	1	-	1	1	1	1	1	1.00 ± 0.00
		Eyes	1	-	0	2	2	1	0	1.00 ± 0.89
		Total Scoring	12	-	8	14	12	7	7	10.00 ± 3.03
3dpi [a]	IN [b]	Body weight	0	0	0	0	0	0	0	0.00
		Appearance	0	0	0	0	0	0	0	0.00
		Motor activity	0	0	0	0	0	0	0	0.00
		Posture	0	1	0	0	0	0	0	0.14 ± 0.38
		Respiration rate	0	0	0	0	0	0	0	0.00
		Eyes	0	0	0	0	0	0	0	0.00
		Total Scoring	0	1	0	0	0	0	0	0.14 ± 0.38
	ITI [c]	Body weight	0	0	0	0	0	-	0	0.00
		Appearance	1	0	0	1	0	-	0	0.33 ± 0.52
		Motor activity	1	1	0	3	1	-	1	1.17 ± 0.98
		Posture	1	0	0	3	1	-	1	1.00 ± 1.10
		Respiration rate	1	0	1	1	0	-	1	0.67 ± 0.52
		Eyes	0	1	0	0	0	-	0	0.17 ± 0.41
		Total Scoring	4	2	1	9	2	-	3	3.50 ± 2.88
	ITA [d]	Body weight	0	-	1	-	1	1	1	0.80 ± 0.45
		Appearance	1	-	2	-	1	1	1	1.20 ± 0.45

		Motor activity	1	-	1	-	2	1	1	1.20 ± 0.45
		Posture	1	-	2	-	2	1	1	1.40 ± 0.55
		Respiration rate	1	-	1	-	1	1	1	1.00 ± 0.00
		Eyes	1	-	0	-	1	0	0	0.40 ± 0.55
		Total Scoring	5	-	7	-	8	5	5	6.00 ± 1.41
4dpi [a]	IN [b]	Body weight	0	0	0	0	0	0	0	0.00
		Appearance	0	0	0	0	0	0	0	0.00
		Motor activity	0	0	0	0	0	0	0	0.00
		Posture	0	0	0	0	0	0	0	0.00
		Respiration rate	0	0	0	0	0	0	0	0.00
		Eyes	0	0	0	0	0	0	0	0.00
		Total Scoring	0	0	0	0	0	0	0	0.00
	ITI [c]	Body weight	0	0	0	-	0	-	0	0.00
		Appearance	0	0	0	-	0	-	0	0.00
		Motor activity	1	1	0	-	1	-	1	0.80 ± 0.45
		Posture	1	0	0	-	0	-	1	0.40 ± 0.55
		Respiration rate	0	0	0	-	0	-	0	0.00
		Eyes	0	0	0	-	0	-	0	0.00
		Total Scoring	2	1	0	-	1	-	2	1.20 ± 0.84
	ITA [d]	Body weight	0	-	0	-	-	0	0	0.00
		Appearance	1	-	1	-	-	1	0	0.75 ± 0.50
		Motor activity	1	-	1	-	-	1	1	1.00 ± 0.00
		Posture	1	-	1	-	-	1	1	1.00 ± 0.00
		Respiration rate	0	-	1	-	-	0	0	0.25 ± 0.50
		Eyes	0	-	0	-	-	0	0	0.00
		Total Scoring	3	-	4	-	-	3	2	3.00 ± 0.82
5dpi [a]	IN [b]	Body weight	0	0	0	0	0	0	0	0.00
		Appearance	0	0	0	0	0	0	0	0.00
		Motor activity	0	0	0	0	0	0	0	0.00
		Posture	0	0	0	0	0	0	0	0.00
		Respiration rate	0	0	0	0	0	0	0	0.00
		Eyes	0	0	0	0	0	0	0	0.00
		Total Scoring	0	0	0	0	0	0	0	0.00
	ITI [c]	Body weight	0	0	0	-	0	-	0	0.00
		Appearance	0	0	0	-	0	-	0	0.00
		Motor activity	0	0	0	-	0	-	0	0.00
		Posture	1	0	0	-	0	-	1	0.40 ± 0.55
		Respiration rate	0	0	0	-	0	-	0	0.00
		Eyes	0	0	0	-	0	-	0	0.00
		Total Scoring	1	0	0	-	0	-	1	0.40 ± 0.55
	ITA [d]	Body weight	0	-	0	-	-	0	0	0.00
		Appearance	1	-	1	-	-	0	0	0.50 ± 0.58
		Motor activity	0	-	1	-	-	0	0	0.25 ± 0.50
		Posture	1	-	1	-	-	1	1	1.00 ± 0.00
		Respiration rate	0	-	0	-	-	0	0	0.00
		Eyes	0	-	0	-	-	0	0	0.00
		Total Scoring	2	-	3	-	-	1	1	1.75 ± 0.96
6dpi [a]	IN [b]	Body weight	0	0	0	0	0	0	0	0.00
		Appearance	0	0	0	0	0	0	0	0.00
		Motor activity	0	0	0	0	0	0	0	0.00
		Posture	0	0	0	0	0	0	0	0.00

		Respiration rate	0	0	0	0	0	0	0	0.00
		Eyes	0	0	0	0	0	0	0	0.00
		Total Scoring	0	0	0	0	0	0	0	0.00
	ITI [c]	Body weight	0	0	0	-	0	-	0	0.00
		Appearance	0	0	0	-	0	-	0	0.00
		Motor activity	0	0	0	-	0	-	0	0.00
		Posture	1	0	0	-	0	-	0	0.20 ± 0.45
		Respiration rate	0	0	0	-	0	-	0	0.00
		Eyes	0	0	0	-	0	-	0	0.00
		Total Scoring	1	0	0	-	0	-	0	0.20 ± 0.45
	ITA [d]	Body weight	0	-	0	-	-	0	0	0.00
		Appearance	0	-	0	-	-	0	0	0.00
		Motor activity	0	-	1	-	-	0	0	0.25 ± 0.50
		Posture	1	-	1	-	-	1	0	0.75 ± 0.50
		Respiration rate	0	-	0	-	-	0	0	0.00
		Eyes	0	-	0	-	-	0	0	0.00
		Total Scoring	1	-	2	-	-	1	0	1.00 ± 0.82
7dpi [a]	IN [b]	Body weight	0	0	0	0	0	0	0	0.00
		Appearance	0	0	0	0	0	0	0	0.00
		Motor activity	0	0	0	0	0	0	0	0.00
		Posture	0	0	0	0	0	0	0	0.00
		Respiration rate	0	0	0	0	0	0	0	0.00
		Eyes	0	0	0	0	0	0	0	0.00
		Total Scoring	0	0	0	0	0	0	0	0.00
	ITI [c]	Body weight	0	0	0	-	0	-	0	0.00
		Appearance	0	0	0	-	0	-	0	0.00
		Motor activity	0	0	0	-	0	-	0	0.00
		Posture	0	0	0	-	0	-	0	0.00
		Respiration rate	0	0	0	-	0	-	0	0.00
		Eyes	0	0	0	-	0	-	0	0.00
		Total Scoring	0	0	0	-	0	-	0	0.00
	ITA [d]	Body weight	0	-	0	-	-	0	0	0.00
		Appearance	0	-	0	-	-	0	0	0.00
		Motor activity	0	-	0	-	-	0	0	0.00
		Posture	0	-	1	-	-	0	0	0.25 ± 0.50
		Respiration rate	0	-	0	-	-	0	0	0.00
		Eyes	0	-	0	-	-	0	0	0.00
		Total Scoring	0	-	1	-	-	0	0	0.25 ± 0.50

[a] dpi: day post infection; [b] IN: intranasal inoculation; [c] ITI: intratracheal instillation; [d] ITA: intratracheal aerosolization; [e] SD: standard deviation.

Table S3. Top 10 GO terms that are significantly enriched in intratracheal aerosolization at 3dpi.

GO ID	Description	Counts	*p*-Value
GO:0005575	cellular component	2650	8.75×10^{-14}
GO:0110165	cellular anatomical entity	2509	1.45×10^{-84}
GO:0008150	biological process	2453	3.38×10^{-11}
GO:0003674	molecular function	2363	3.01×10^{-5}
GO:0009987	cellular process	1931	1.37×10^{-25}
GO:0005488	binding	1870	4.12×10^{-46}
GO:0065007	biological regulation	1631	1.49×10^{-118}
GO:0050789	regulation of biological process	1577	1.77×10^{-111}
GO:0050794	regulation of cellular process	1473	9.21×10^{-92}

GO:0016020	membrane	1293	5.27×10^{-58}

Table S4. Top 10 KEGG terms that are significantly enriched in intratracheal aerosolization at 3dpi.

**Pathway ID**	**Description**	**Counts**	***p*-Value**
mmu05200	Pathways in cancer	103	6.56×10^{-4}
mmu04060	Cytokine-cytokine receptor interaction	95	2.69×10^{-15}
mmu04151	PI3K-Akt signaling pathway	79	2.19×10^{-5}
mmu05165	Human papillomavirus infection	72	6.53×10^{-4}
mmu04814	Motor proteins	58	1.67×10^{-8}
mmu04621	NOD-like receptor signaling pathway	58	3.12×10^{-7}
mmu05022	Pathways of neurodegeneration-multiple diseases	58	1.31×10^{-4}
mmu05132	Salmonella infection	56	2.01×10^{-4}
mmu04380	Osteoclast differentiation	55	1.11×10^{-13}
mmu04062	Chemokine signaling pathway	54	5.55×10^{-7}
